# Insights on the microbiology of Ethiopian fermented milk products: A review

**DOI:** 10.1002/fsn3.4372

**Published:** 2024-08-01

**Authors:** Tiruha H. Karssa, Jamal B. Kussaga, Teresa Semedo‐Lemsaddek, Jovin K. Mugula

**Affiliations:** ^1^ Department of Biology Hawassa University Hawassa Ethiopia; ^2^ Department of Food Science and Agro‐Processing Sokoine University of Agriculture Morogoro Tanzania; ^3^ CIISA – Centre for Interdisciplinary Research in Animal Health, Faculty of Veterinary Medicine University of Lisbon Lisbon Portugal; ^4^ Associate Laboratory for Animal and Veterinary Sciences (AL4AnimalS) Lisbon Portugal; ^5^ BioISI – Biosystems & Integrative Sciences Institute, Faculty of Sciences, University of Lisbon Lisbon Portugal

**Keywords:** Ethiopia, lactic acid bacteria, microbiology, traditional fermented milk products, yeasts

## Abstract

Fermented milk products play a vital role in the diets of Ethiopians. They are produced from either spontaneous fermentation or back‐slopping methods at the household level, in which lactic acid bacteria (LAB) and yeasts predominate. As a result, the processing steps are not standardized and overall safety is still of public health relevance. Therefore, quality and safety improvement, standardization of traditional manufacturing practices, and commercialization of products to a wider market are important. Hence, this systematic review aimed to provide a comprehensive overview of the microbiology of traditional Ethiopian fermented milk products, including *ergo* (spontaneously fermented whole milk), *dhanaan* (fermented camel milk), *ititu* (concentrated sour milk or spontaneously fermented milk curd), *ayib* (traditional cottage cheese), *qibe* (traditional butter), *arrera* (defatted buttermilk), and *hazo* (spiced fermented buttermilk). We followed the Preferred Reporting Items for Systematic Reviews and searched relevant databases and search engines, including the Web of Science, Google Scholar, Scopus, PubMed, ScienceDirect, and ResearchGate. Furthermore, the pertinent literature was checked individually and identified. Dairy fermentation provides shelf‐life extension and improves the organoleptic quality of products. Nonetheless, the aforementioned Ethiopian fermented foods may be contaminated with *Escherichia coli* 0157: H7, *Listeria monocytogenes*, *Salmonella* spp., or *Staphylococcus aureus* due to inadequate processing and handling practices. This systematic review also revealed that these traditional milk products lack consistent quality and safety due to poor hygienic preparation techniques, non‐controlled fermentation, and limited knowledge or awareness of small‐holder dairy farmers. Therefore, the use of suitable procedures including good hygienic practices and controlled fermentation is recommended.

## Graphical abstract

Dairy fermentation provides shelf‐life extension and improves the organoleptic quality of products. Nonetheless, Ethiopian fermented foods may be contaminated with *Escherichia coli* 0157: H7, *Listeria monocytogenes*, *Salmonella* spp., or *Staphylococcus aureus* due to inadequate processing and handling practices. This systematic review also revealed that these traditional milk products lack consistent quality and safety due to poor hygienic preparation techniques, non‐controlled fermentation, and limited knowledge or awareness of small‐holder dairy farmers.

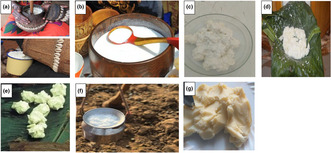

## 1 INTRODUCTION

Milk products are an integral part of the diet of small holder farmers in Africa, playing a vital role in the enhancement of food security and income generation in both rural and urban communities. In Ethiopia, a significant amount of milk is consumed in fermented form, because of its longer shelf life and associated health benefits.

Ethiopia has the largest livestock population in Africa (approximately 170 million) and is the tenth‐largest in the world (CSA (Central Statistical Authority), 2021). According to the Ethiopian Central Statistical Authority (CSA, 2021), the country has 65.35, 39.89, 50.50, 2.11, 8.98, 0.38, and 7.70 million heads of cattle, sheep, goats, horses, donkeys, mules, and camels, respectively. The dairy sector is one of the oldest livestock sub‐sectors and is characterized by rural smallholders with indigenous cattle (Duguma, 2022).

Although camels, goats, and to a lesser extent sheep are used for milk production, approximately 90% of the milk is obtained from cattle (Getahun et al., 2019; Gonfa et al., 2001). Despite a large livestock population, milk production remains low. Milk products constitute an important component of the human diet in Ethiopia. A large proportion of milk is consumed in products prepared using traditional manufacturing methods (Getahun et al., 2019).

Fermented milk products have distinct vernacular names in different parts and various ethnic groups in the country, including *ergo* (sour milk), *dhanaan* (fermented camel milk), *ititu* (milk curd), *ayib* (cottage cheese), *neter kibe* (spiced butter), *kibe* (traditional butter), *aguat* (whey), and *arerra* (sour defatted milk) (Andualemm & Geremew, 2014; Ashenafi, 2008; Getahun et al., 2019). They are spontaneously fermented without the addition of defined starter cultures. Proliferation of the initial microbiota initiates fermentation, with microbial succession being influenced by room temperature and biochemical changes in the fermenting milk (Andualemm & Geremew, 2014; Ashenafi, 2008; Berhe, 2017). Lactic acid bacteria (LAB) and yeasts predominate during fermentation process (Berhe, 2017). Although the products undergo similar processing steps, a lack of control of processing conditions may promote the proliferation of undesirable microorganisms, which may compromise the overall product's quality and safety. This indicates that milk products of inconsistent quality were produced.

Berhe, 2017 reviewed the traditional fermented dairy products of Ethiopia, focusing on indigenous processing practices, and found that their production is substandard. These authors also discussed alternative strategies to standardize manufacturing practices, for the improvement of milk product quality and safety. Similarly, Gonfa et al. (2001) reviewed the variety and socio‐economic and dietary importance of traditional fermented milk products in Ethiopia, aiming to document traditional technologies and information on microbiology, leading to the identification of various constraints on the development and commercialization of fermented milk products.

Frew and Abebe (2020) reviewed the microbial properties of milk products produced in different parts of Ethiopia. The main factors affecting microbial properties of milk products are discussed, and the respective microorganisms of some common milk products are presented. Additionally, Andualemm and Geremew (2014) studied beneficial microorganisms in Ethiopian fermented milk products. The authors reported that lactic acid bacteria are the dominant microorganisms in dairy products. The role of lactic acid bacteria in food preservation, and their antagonistic effects against foodborne pathogens, were investigated. Getahun et al. (2019) highlighted an overview of probiotics in relation to the Ethiopian dairy industry and reported lactic acid bacteria as potential probiotics that play an essential role in fermenting milk products, as well as preventing the growth of spoilage microorganisms.

Various studies have also reviewed other Ethiopian fermented foods such as *enjera* (Mengesha et al., 2022), *kocho* (Karssa et al., 2014), *wakalim* (Ketema et al., 2009), *borde* (Abegaz et al., 2002), *tella* (Tekle et al., 2019), *cheka* (Worku et al., 2018), *keribo* (Dibaba et al., 2018), and *shamita* (Kitessa et al., 2022), but there is little comprehensive review of the literature on the microbiology and safety of traditional dairy products. Lack of information undermines stakeholders' efforts to develop interventions along the product value chain. Therefore, this review aimed to provide a comprehensive insight into the microbiology of traditional Ethiopian fermented milk products. Additionally, the variety and commercial potential of traditional fermented milk products in Ethiopia, along with associated beneficial microorganisms, including lactic acid bacteria and yeasts, as well as pathogenic microorganisms, including *E. coli* O157:H7, *Listeria monocytogenes*, *Salmonella*, and *Staphylococcus aureus*, are discussed in this review paper. Finally, concluding remarks and future directions are proposed for the development of the dairy sector and scale‐up of production.

## 2 METHODOLOGY

This systematic review was conducted according to the Preferred Reporting Items for Systematic Reviews and Meta‐Analyses (PRISMA) protocols.

### 2.1 Database search

Data published on fermented milk products were gathered from various sources using relevant databases and websites, including Web of Science, Google Scholar, Scopus, PubMed, ScienceDirect, and ResearchGate, using key terms including fermentation, milk products, microbiology, probiotics, lactic acid bacteria, and yeasts. A database search was performed individually by one of the researchers (THK), and articles were identified and selected for pooling them together later. Various key search terms were combined using Boolean operators like “AND” and “OR” to obtain relevant articles. We used all possible combinations of the following keywords in our search strategy: “Fermented milk” AND “probiotic bacteria” AND “yeast”; “fermented milk” OR “ergo” AND “dhanaan” AND “ititu”; “fermented milk product” OR “ayib” AND “quibe” AND “arrera” AND “Hazo”; “fermented milk product” AND pathogenic microorganisms; “fermented milk product” AND “commercial potential.” We manually scanned the reference lists of pertinent papers to find further studies that might have been overlooked during the database search. All selected relevant articles were exported to Mendeley Desktop reference management software for appropriate citation and referencing. A thorough overview of the most recent findings was provided by integrating the data from various sources. Therefore, the information presented in this review provides an important scientific foundation for future studies on traditional fermented milk products.

### 2.2 Study selection

Following the initial stage of the screening process, which involved removing duplicates using Mendeley reference manager, titles and abstracts of the remaining articles were evaluated in order to identify relevant articles. In the second stage, the full texts of the papers were examined if the abstracts did not provide enough information to determine their eligibility. Finally, this systematic review included all pertinent articles meeting the inclusion criteria.

### 2.3 Articles inclusion criteria

The titles and abstracts of the articles were evaluated for their relevance to the review. After assessing the titles and abstracts, the inclusion criteria were used to determine which article should be included in the review. We considered articles that were published in peer‐reviewed journals, written in English language, full‐text available, and those describing the microbiology of Ethiopian fermented milk and other related products. Additionally, studies focusing on beneficial and pathogenic microorganisms in fermented dairy products have been included. After the initial screening process, 311 articles were selected and exported into Mendeley Desktop reference management software for appropriate citation and referencing of this systematic review.

### 2.4 Articles exclusion criteria

**FIGURE 1:**
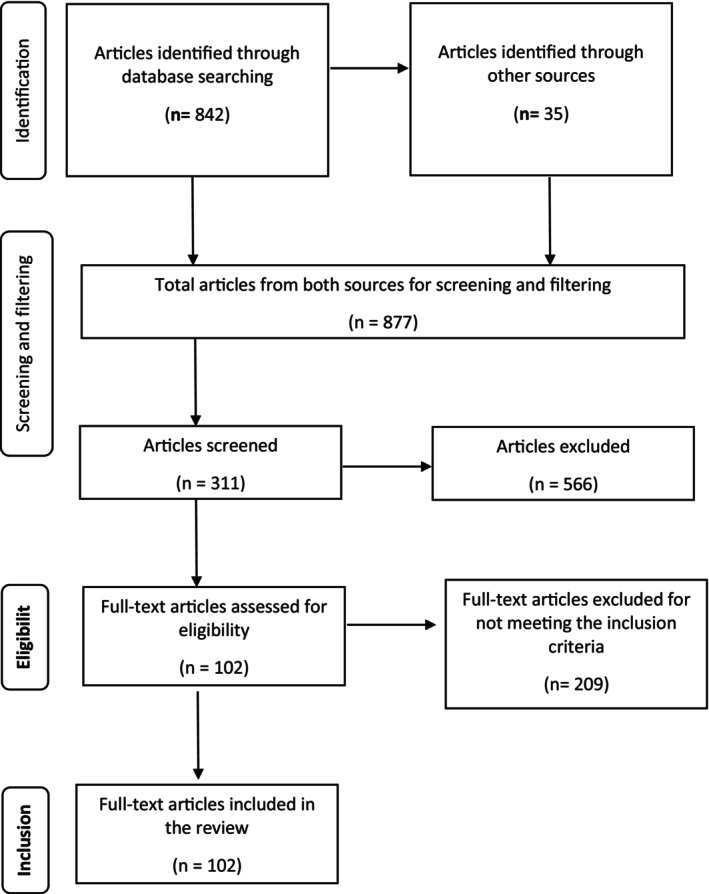
PRISMA flowchart of searching, screening, eligibility, and inclusion of articles for the systematic review.

Articles that were irrelevant to the contents of this review, non‐peer‐reviewed, not written in English language, with no full text available, and those with insufficient information about the microbiology of Ethiopian fermented milk and other related products were excluded. Additionally, old literature was excluded, with a few exceptions. Following the Preferred Reporting Items for Systematic Reviews (PRISMA) (Figure 1), after searching, identifying, screening, and filtering processes, 102 relevant articles were selected and included in this systematic review.

## 3 FERMENTED MILK PRODUCTS IN ETHIOPIA

### 3.1 Ergo

**FIGURE 2:**
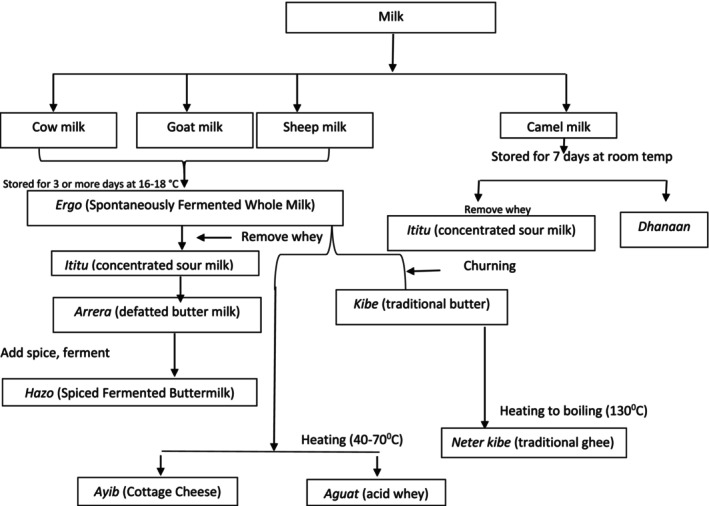
Flowchart of processing steps of different traditional fermented Ethiopian milk products (Berhe, 2017).

**FIGURE 3:**
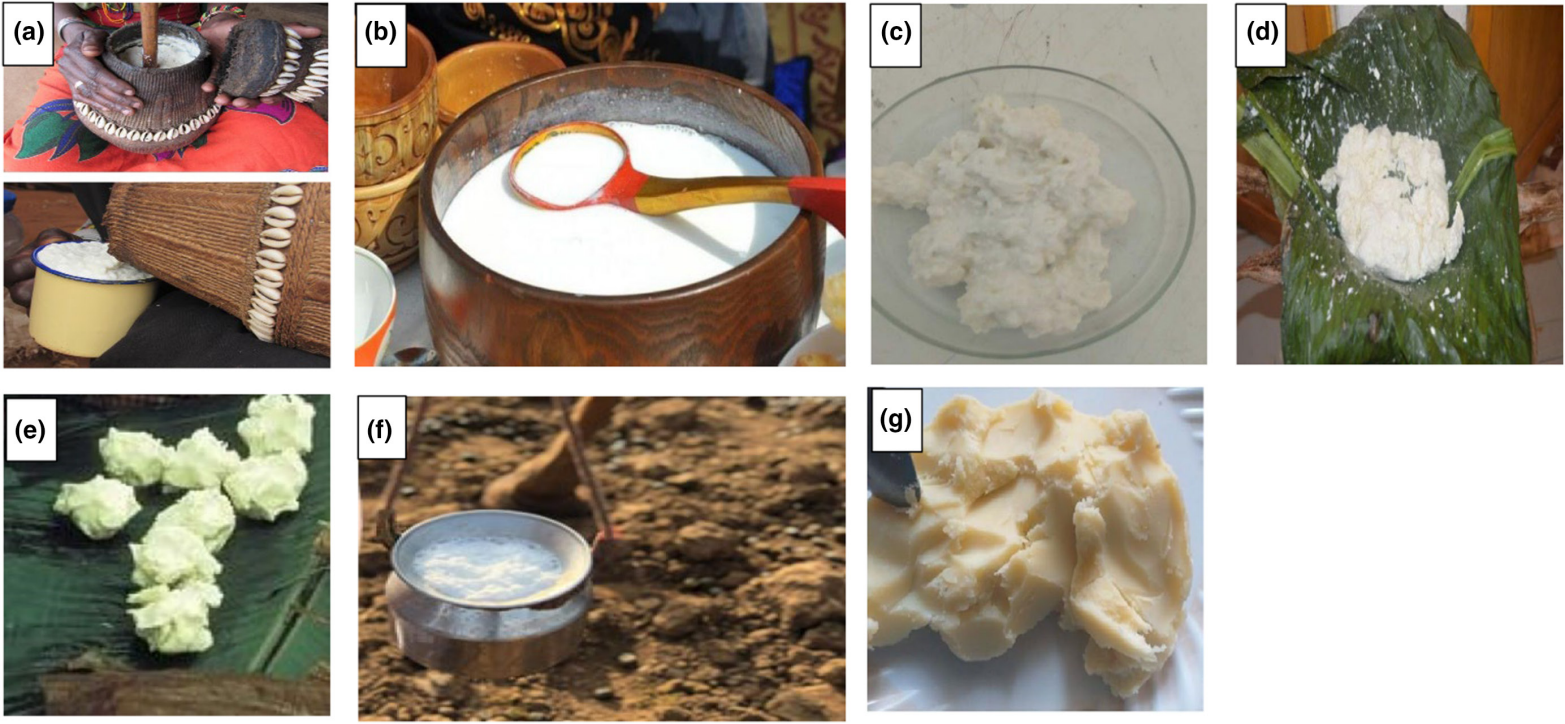
Traditionally fermented milk products of Ethiopia. (a) *Ergo* (spontaneously fermented whole milk), (b) *Dhanaan* (fermented camel milk), (c) *Ititu* (concentrated sour milk), (d) *Ayib* (cottage cheese), (e) *Qibe* (traditional butter), (f) *Arrera* (defatted buttermilk), (g) *Neter kibe* (traditional ghee). Amenu et al. (2019), Hussien (2020), Kuyu and Bereka (2020).

*Ergo* is a spontaneously fermented milk product mainly prepared at the house level. In *ergo* production, raw milk is left at an ambient temperature of 16–18°C for 24–72 h (Frew & Abebe, 2020) for fermentation. In rural areas, raw milk is usually placed in well‐smoked clay pots (*Ensira*), especially among pastoralists, and fermentation is carried out spontaneously and/or by back‐slopping. LAB are the dominant microorganisms, followed by yeasts and molds. Yeasts are known to contribute to the flavor of the fermented milk, whereas some mold species serve as adjunct cultures for the production of fermented milk (Šipošová et al., 2021). Depending on the room temperature (25–35°C), the product has a shelf‐life of 15–20 days (Andualemm & Geremew, 2014). The fermented product can also be processed into traditional butter (*qibe*) or buttermilk (*arrera*). Buttermilk may further be processed into traditional cottage cheese (*ayib*) and whey (*aguat*) (Figures 2 and 3).

### 3.2 Dhanaan

*Dhanaan* is common in the Somalia region and *Ititu* in the Afar/Oromia region (Berhe, 2017; Seifu et al., 2012). *Dhanaan* is the major fermented camel milk in rural and urban settlements in the Somali Regional State (Biratu & Seifu, 2016; Birhanu et al., 2021). According to Berhe (2017), the traditional production of *dhanaan* is based on the spontaneous fermentation of camel milk at ambient temperature (25–35°C) for approximately 12–24 h. Fresh camel milk was placed in a clean and smoked container with glowing splinters of *Olea africana*, wrapped in a piece of cloth, and maintained at room temperature. The fermentation process is initiated by back‐slopping (Berhe, 2017). Seifu (2007) reported that pastoralists in Somali Regional State produced *dhanaan* due to its perceived high nutritional value, better taste, and longer shelf life. It has been reported that *dhanaan* has a shelf life of five months (Tekle et al., 2019). The physicochemical and nutritional properties of *dhanaan* include pH (4.18), titratable acidity (1.8%), total protein (4.1%), fat (2.5%), total solids (11.1%), solids other than fat (8.6%), and ash content (1.0%) (Biratu & Seifu, 2016). Camel milk α‐lactalbumin has higher digestibility by pancreatic proteases and more antioxidant activity than bovine α‐lactalbumin (Salami et al., 2009). This suggests a potential benefit of camel α‐lactalbumin for use in infant food formulations. Camel milk is similar to human milk in the absence of β‐lactoglobulin, which may cause allergies (El‐Agamy et al., 2009).

### 3.3 Ititu

*Ititu* is concentrated fermented milk prepared and consumed by the Borana community in southern Ethiopia and *kereyu* area of the Oromia region in eastern Ethiopia (Andualemm & Geremew, 2014; Hussien, 2020; Seifu et al., 2012). *Ititu*, like *ergo*, is commonly spontaneously produced from raw camel milk (Berhe, 2017). However, it can also be prepared using cow, goat, or sheep milk (Seifu et al., 2012). In its preparation, fresh milk is collected in a fermenting vessel called *Gorfa*, which is mainly smoked with *Olea africana* and allowed to ferment at 27–30°C for 15–30 days (Figures 2 and 3). The periodic removal of whey from fermented milk and addition of fresh milk at interval of 3–4 days are the main steps in the production of *Ititu* (Berhe, 2017; Hussien, 2020). These steps are performed several times until the product is concentrated and ready for consumption (Hussien, 2020). *Ititu* has a high nutritional quality, medicinal properties, and shelf life of 2–3 months at room temperature (Gonfa et al., 2001; Mulaw and Tesfaye, 2017). Traditionally prepared *Ititu* shows average values of pH, titratable acidity, total protein, fat, total solids, and ash 3.59 ± 0.04, 2.86% ± 0.18%, 7.26% ± 0.41%, 9.85% ± 0.73%, 21.23% ± 1.48%, and 0.84% ± 0.11%, respectively (Hussien et al., 2021). *Ititu* has a higher free and total amino acid content than fresh milk (Hussien, 2020). Moreover, it is rich in non‐essential amino acids, such as glutamic acid, alanine, proline, and serine (Mulaw and Tesfaye, 2017).

### 3.4 Ayib

*Ayib* is a traditional Ethiopian cottage cheese made from defatted sour milk (*arerra*) after the fat is removed by churning. The milk to be fermented is collected in a clay pot and kept in a warm place (approximately 30°C) for 24–48 h to sour spontaneously (Ashenafi, 2002). The sour milk is churned by gradually shaking the contents of the pot until the fat separates. The fat is then removed and the defatted milk is heated in a clay pot to approximately 50°C, until a distinct curd mass is formed and floats over the whey, followed by cooling to coagulate and recover the curd from the whey (Mossie, 2019). Finally, the curd is separated from the whey using a fine mesh cloth or sieve and is kept in a clean bowl or pot (Ayenew et al., 2009; Kuyu & Bereka, 2020).

*Ayib* is an important source of nutrients and serves as a staple food in various parts of Ethiopia. It is comprised of water, protein, fat, ash, and soluble milk constituents of 79%, 14.7%, 1.8%, 0.9%, and 3.1%, respectively (Mulaw and Tesfaye, 2017). It is consumed fresh as a condiment, or with hot spices, salt, and herbs (Gonfa et al., 2001; Hailemikael Mossie, 2019). Spices, salt, and herbs, including *Koserete* (*Ocimum hardiense*), *Tikure azmude* (*Nigella sativa*), *Korerima* (*Aframomum angustifolium*), *Tena Adam* (*Ruta chalepensis*), and *Abish* (*Trigonela fenum*), are added to extend shelf life and improve sensory features (Abebe et al., 2014; Eshetu & Asresie, 2019).

### 3.5 Qibe

*Qibe* is a traditional Ethiopian butter that is produced by churning *ergo* (fermented whole milk). Milk intended for churning is accumulated over several days by the continuous addition of fresh milk to the already accumulated fermented milk. When a sufficient amount of milk is collected and fermented into *ergo*, it is filled into a traditional churner (clay pot). The churner is then agitated back and forth after covering the mouth of the churner tightly with materials such as the pseudo‐stem of *enset* (*Ensete ventricosum*) (Berhe, 2017). At the end of churning, small fat granules coalesce into larger grains and form lumps of fat grains at the base of the churn and are finally removed. The butter is then kneaded in cold water and washed to remove visible residual buttermilk (Kuyu & Bereka, 2020). Women are responsible for the entire process of churning and butter making.

In addition from human consumption, fresh butter is used by women as a traditional cosmetic product for skin and hair (Andualemm & Geremew, 2014). It is sold at much a higher price than other milk products, and is an important source of income for households. Fresh butter can be processed into *nitir qibe* (traditional ghee) by adding spices and herbs and then melted by heating prior to consumption.

### 3.6 Arrera

*Arrera* is a semi‐liquid product that remains after butter making. It contains casein and whey with a thin and smooth consistency (Abebe et al., 2014). It has a similar color, taste, and aroma to *ergo*. It may be consumed as it is (fresh) or further processed into *ayib* by heating. Fresh *arrera* is consumed as complementary food by children and the elderly in low‐income households in rural areas. If there is a surplus, it can be fed to calves, lactating cows, and dogs (Gonfa et al., 2001). *Arrera* is known to have fewer calories and a shorter shelf life 24–48 h than other traditional milk products. *Arrera* contains moisture 91.5%, protein 3.1%, fat 1.4%, carbohydrate 3.4%, and ash 0.6% (Zahara & Zinewi, 2015).

### 3.7 Hazo

*Hazo* is a traditionally fermented milk product prepared from defatted buttermilk (*arrera*), with the addition of pulses or cereal grain flour and spices, such as pepper and garlic. It is then fermented for 2–3 days at room temperature (Gebreselassie et al., 2012). It is most common in the northern part of Ethiopia, particularly in the Tigray Regional State. The final product is reddish and has thicker consistency than buttermilk. It served for guests, respected family members, and during socio‐cultural festivals called *hazo* (Berhe, 2017; Negash, 2018). It has a shelf life of 1–2 weeks with a possible extension of up to one month, through the addition of a newly fermented *hazo* every week (Gebreselassie et al., 2012). *Hazo* has various claimed health benefits, owing to the addition of herbs and spices.

## 4 BENEFICIAL MICROORGANISMS OF FERMENTED DAIRY PRODUCTS

A wide variety of microbes are involved in milk fermentation. LAB and yeasts are major fermenting microorganisms. Both LAB and yeast have symbiotic relationships during fermentation. LAB produce acid, which promotes yeast proliferation, whereas yeasts provide vitamins and other growth factors that are used by LAB. LAB can acidify milk and contribute to other organoleptic attributes during production of fermented milk products.

### 4.1 Lactic acid bacteria

LAB are the predominant microorganisms in fermented milk products. These bacteria are generally regarded as safe (GRAS) by the US Food and Drug Administration (FDA) and harbor Qualified Presumption of Safety (QPS) by the European Food Safety Authority (EFSA). They are widely used for food fermentation on a global scale (Getahun et al., 2019). These microbial groups comprise several genera, including *Bifidobacterium*, *Streptococcus*, *Enterococcus*, *Lactococcus*, *Leuconostoc*, *Lactobacillus*, *and Pediococcus*. Furthermore, *Lactobacillus*, *Lactococcus*, *Leuconostoc*, *Pediococcus*, *and Streptococcus* are widely known to be industrially important microorganisms for the production of milk products (Teshome, 2015).

#### 4.1.1 Lactic acid bacteria in food preservation

LAB have been used for bio‐preservation of food, at the household level in many countries, particularly developing countries that lack high‐level facilities and infrastructure. Some LAB produce bacteriocins, which are considered valuable substitutes for chemical preservatives in food production systems, contributing to the safe and effective natural inhibition of pathogenic and spoilage bacteria (Leroy & De Vuyst, 2010; Sharma et al., 2021; Zapaśnik et al., 2022). In addition, during fermentation, LAB produce a wide range of metabolites with antimicrobial activity including lactic acid, acetic acid, hydrogen peroxide, and other low molecular weight substances (reuterin, diacetyl, reutericyclin, and fatty acids) and antifungal compounds (phenyl lactate, propionate, and hydroxyphenyl lactate) (Ibrahim et al., 2021). LAB fermentation also contributes to the sensory features (flavor, aroma, and texture) of fermented products and improves the digestibility and nutritional quality of foods (González‐González et al., 2022).

#### 4.1.2 Functional lactic acid bacteria starter cultures

Starter cultures with a large number or concentration of microorganisms can comprise a single type or mixture of two or more microorganisms. They are added to food materials in order to take advantage of the compounds or products derived from their metabolism or enzymatic activity, and to bring about desired and predictable changes in the finished product. These changes may include enhanced preservation, improved nutritional quality, modified sensory qualities, and increased economic value (Durso & Hutkins, 2003).

Although many fermented foods can be manufactured without external starter cultures, the addition of concentrated microorganisms, in the form of starter culture, provides a basis for ensuring that these products are manufactured on a reliable schedule, with consistent qualities (Durso & Hutkins, 2003). The most promising microorganisms selected as starter culture are those that are isolated from the native autochthonous microbiota of traditional products, since they are well adapted to the environmental conditions of food and are capable of controlling spoilage and pathogenic microbiota.

Starter cultures with at least one inherent functional characteristic are considered functional. Functional starter cultures enhance food safety and/or provide organoleptic, technological (high production), nutritional, or health advantages (Table 1). LAB play key roles in fermentation processes and have a long history of application and safe production of fermented foods and beverages worldwide (Abiola et al., 2022).

**TABLE 1:** LAB functional starter cultures and associated role in Ethiopian fermented foods.

Type of fermented product	Functionality	LAB genus/species	References
Fermented cereals			
Rice *injera*	Nutritional, shelf life, and sensory quality	*Lactiplantibacillus plantarum* and *Limosilactobacillus fermentum*	(Hassen et al., 2018)
*Teff injera*	Folate production	*Lactiplantibacillus plantarum*	(Tamene et al., 2022)
*Teff* (*Eragrostis tef*)	Fermentation, sensory quality, and safety	*Lacticaseibacillus paracasei*, *Levilactobacillus brevis*, *Enterococcus durans*, *Enterococcus hirae*, *Enterococcus avium*, and *Enterococcus faecium*	(Dibaba et al., 2018)
*Shameta*		*Lactobacillus*	(Kitessa et al., 2022)
Fermented milk products			
*Ititu*	Fermentation	*Ligilactobacillus salivarius*	(Seifu et al., 2012)
*Dhanaan*	Fermentation	*Streptococcus*, *Weissella*, and *Lactococcus*	(Tekle et al., 2019)
*Ergo*	Sensory quality, shelf life, and safety	*Lacticaseibacillus paracasei*, *Lacticaseibacillus rhamnosus*, *Lactiplantibacillus plantarum*, *Latilactobacillus sakei*, *Latilactobacillus curvatus*, and *Lacticaseibacillus casei*	(Asefa et al., 2022)
Buttermilk	Fermentation	*Lactococcus lactis* and *Lactiplantibacillus plantarum*	(Gebreselassie et al., 2016)
Fermented plant products			
*Enset* (*Ensete ventricosum*)	Fermentation	*Lactiplantibacillus plantarum* and *Leuconostoc mesenteroides*	(Andeta et al., 2019)

#### 4.1.3 Probiotic potential of lactic acid bacteria

Probiotics are microorganisms that, when administered in adequate amounts, confer health benefits to consumers (Bieckale, 2006). Currently, it is used to refer to bacteria that confer beneficial effects on both humans and animals. Probiotics have several health benefits for humans, including balancing the gut microbiota and protection against pathogens. Probiotics have also been reported to stimulate the immune system, and lower blood cholesterol levels, vitamin synthesis, and anticarcinogenic properties (Markowiak & Ślizewska, 2017; Yerlikaya, 2019). Several fermented dairy products harbor probiotic microorganisms. The consumption of fermented foods, including milk products such as *dhanaan*, yogurt, and cheese, has immense potential to improve human health and well‐being, through stimulating the actions of the gut microbiota via the production of bioactive chemicals and neuropeptides (Mokoena et al., 2016).

Diverse probiotic bacteria have been explored in the food and dairy industries, with the goal of producing goods with excellent health benefits at a low cost (Ayivi & Ibrahim, 2022). Human LAB probiotics mainly belong to the genera *Lactobacillus*, *Lactococcus*, *Bifidobacterium*, *Streptococcus*, and *Enterococcus* (Markowiak & Ślizewska, 2017; Yasmin et al., 2020). The most widely used and commercially available representative species include *Lactiplantibacillus plantarum*, *Lacticaseibacillus casei*, *Lactobacillus johnsonii*, *Lacticaseibacillus rhamnosus*, *Lactobacillus gasseri*, *Bifidobacterium bifidum*, and *Bifidobacterium infantis* (Das et al., 2022; Mulaw et al., 2019; Soccol et al., 2010; Yerlikaya, 2019). *Pediococcus acidilactici* isolated from traditional Ethiopian cereal‐based fermented beverage, “Borde,” has been found to be the best probiotic strain for cholesterol reduction (Gebre et al., 2023). Moreover, some species of probiotic lactic acid bacteria, including *Lactiplantibacillus plantarum*, *Lactiplantibacillus pentosus*, and *Pediococcus pentosaceus*, have been isolated from Ethiopian commercial and spontaneously fermented cheeses (Gizachew et al., 2023). These authors also assessed the antibacterial and immunostimulatory activities of the isolates and confirmed their promising antimicrobial activity against foodborne pathogens and immunomodulatory properties. Probiotic LAB associated with traditional fermented foods can also be used as bio‐preservatives through the production of antimicrobial compounds such as bacteriocins, organic acids, and hydrogen peroxide (Hotessa & Robe, 2020). They also produce nutraceuticals, and thereby increasing the bioavailability of nutrients and providing additional benefits. In addition, probiotic LAB from *Metata Ayib* (traditional spiced cottage cheese) have been found to harbor high probiotic potential and have been suggested for use in further research and development as food and/or feed additives (Adugna & Andualem, 2023). Various LAB species from traditional Ethiopian fermented beef sausage “Wakalim” have also shown promising probiotic potential with health‐promoting effects (Ketema et al., 2009).

### 4.2 Yeasts

Yeasts play a significant role in improving the sensorial and nutritional aspects of fermented products through enzymatic cleavage, metabolite production, and bio‐functionalities (Annunziata et al., 2020; Tamang & Lama, 2022). Some yeast species have also demonstrated probiotic properties (Lara‐Hidalgo et al., 2017; Tamang & Lama, 2022). Yeasts co‐occur with LAB during the natural fermentation of various foods, demonstrating their co‐metabolic activities. Yeasts provide vitamins, amino acids, and other growth factors for LAB, and LAB create an acidic environment conducive to yeast proliferation (Behbahani, 2013; Ewuoso et al., 2020; Johansen et al., 2019).

#### 4.2.1 Yeast functional cultures

Various yeast species play key roles in the functionality of various fermented foods (Table 2). The most common functional properties of yeasts include production of alcohol, metabolites, leavening, and amylolytic enzymatic activities (Tamang & Lama, 2022). Yeasts are notable for the production of flavor compounds by conversion of carbohydrates, particularly in the brewing industry (Johansen et al., 2019). Some yeast species, including *Saccharomyces cerevisiae*, *Pichia kudriavzevii*, *Hanseniaspora guilliermondii*, *Pichia kluyveri*, and *Candida tropicalis*, have been reported to exhibit phytase activity, thereby enhancing the nutrient's bioavailability (Greppi et al., 2015). Nutraceutical compounds that constitute purified food ingredients and offer health benefits are also produced by yeast (Rai et al., 2019). Various genera are only associated with specific fermented products for example, *Saccharomyces* is the most commonly found genus in alcoholic beverages, *Kluyveromyces* in fermented dairy products (Lore et al., 2005). *Pichia* in fermented vegetables; *Metschnikowia*, *Wickerhamomyces* and *Candida* in fermented fish products; *Debaryomyces* in fermented meat and cereal products; and *Torulaspora* in fermented legume products (Tamang & Lama, 2022).

**TABLE 2:** Yeast functional starter cultures and associated role in Ethiopian fermented foods.

Type of fermented product	Functionality	Yeast genus/species	References
Fermented cereals			
Rice *injera*	Nutritional, shelf‐life, and sensory quality	*Saccharomyces cerevisiae*	(Hassen et al., 2018)
*Teff injera*	Folate production	*Saccharomyces cerevisiae*	(Tamene et al., 2022)
Fermented milk products			
*Ergo*	Sensory quality and preservation	*Saccharomyces cerevisiae*	(Asefa et al., 2022)
Buttermilk	Fermentation	*Saccharomyces cerevisiae* and *Kluyveromyces marxianus*	(Gebreselassie et al., 2016)
Fermented plant products			
*Enset* (*Ensete ventricosum*)	Fermentation	*Saturnispora silva*	(Birmeta et al., 2019)

#### 4.2.2 Yeasts as potential probiotics

Probiotic yeasts have been studied less than potential probiotic lactic acid bacteria. Yeasts are promising potential probiotics as they can survive at lower pH values (as low as 1.5) (Tamang & Lama, 2022), and are resistant to antibiotics with no risk associated with horizontal gene transfer events, in comparison with bacterial probiotics (Hsiung et al., 2021). *Saccharomyces cerevisiae* and *Saccharomyces boulardii* are the most common yeast probiotics and have been extensively studied and commercialized to date (de Souza et al., 2022; Hsiung et al., 2021; Lara‐Hidalgo et al., 2017; Tamang & Lama, 2022). It has been reported that when live cultures of *Saccharomyces cerevisiae* are formulated with animal feeds, the growth performance, health, and immune response of nursery pigs are improved (Lara‐Hidalgo et al., 2017). Moreover, several other yeast species, including *Pichia fermentase*, *Pichia kudriavzevii*, *Torulaspora delbrueckii*, *Candida krusei*, *Debaryomyces hansenii*, *Yarrowia lipolytica*, *Kluyveromyces lactis*, and *Kluyveromyces marxianus*, have been shown to have probiotic properties (Rai et al., 2019). Moreover, yeast species with probiotic features have been reported in different types of fermented food (Table 3).

**TABLE 3:** Probiotic yeast species from different fermented foods and beverages.

Fermented product	Probiotic yeast species	Probiotic attributes	References
Dairy products			
		Antioxidant activity	(Goktas et al., 2021; Hsiung et al., 2021)
Kefir	*Kazachstania unispora*	β‐Galactosidase activity	
	*Kazachstania turicensis*	Low pH and bile salt tolerance	
	*Kluyveromyces marxianus*	Auto‐aggregation, hemolysis, and adhesion	
	*Issatchenkia orientalis*, *Pichia kudriavzevii*, *Candida xylopsoci*, and *Saccharomyces cerevisiae*	Low pH and bile salt tolerance, auto‐aggregation, adhesion, and suppression of pathogen	(Basavaiah et al., 2019; Diosma et al., 2014)
Cheese	*Pichia fermentans* *Kluyveromyces marxianus*	Auto‐aggregation, antibacterial, antioxidant, and adhesion	(Banik et al., 2019; Merchán et al., 2020)
Kimchi	*Candida metapsilosis*	Auto‐aggregation, antibacterial, antioxidant, and adhesion	(Hsiung et al., 2021)
Cereals			
Sourdough	*Saccharomyces cerevisiae*	Phytase activity	(Palla et al., 2020; Vuyst et al., 2021)
Tubers			
Cassava	*C. ethanolica*, *G. geotrichum*	Phytase activity	(Moslehi‐Jenabian et al., 2010)

## 5 PATHOGENIC MICROORGANISMS IN FERMENTED MILK PRODUCTS

Although fermented milk products have several therapeutic benefits, but poor handling practices along the value chain can cause contamination with pathogenic microorganisms. Various foodborne illnesses have been implicated in fermented dairy product productions. Common pathogens reported in dairy products include *Escherichia coli* O157:H7, *Listeria monocytogenes*, *Salmonella* spp., and *Staphylococcus aureus* (Yilma et al., 2015).

*Escherichia coli* O157:H7 causes severe intestinal infections in humans (Yilma et al., 2015). It survives milk fermentation and has been detected in various traditional fermented milk products, including *ergo*, *ayib*, butter, and buttermilk (Ogwaro et al., 2023; Yilma et al., 2007). Several *E. coli* O157:H7 outbreaks have been associated with the consumption of milk products (Seifu et al., 2012). Cattle are a reservoir of *E. coli* O157:H7; however, proper processing practices, such as heat treatment and controlled fermentation, may completely destroy the pathogen. *E. coli* 0157: H7 survived at 50 to 60°C in heat‐defatted buttermilk during the production of *ayib* (cottage cheese) (Yilma et al., 2015). However, complete inhibition of *E. coli* O157:H7 can be achieved by cooking defatted buttermilk at 70°C (Tsegaye & Ashenafi, 2005; Yilma et al., 2015). Acid‐adapted *E. coli* O157:H7 may also survive the processing and storage of fermented milk products (Dlamini & Buys, 2009). Therefore, spontaneous fermentation without any thermal treatment, may, not guarantee the safety of the products and could be a public health concern. Regarding Ethiopian milk fermented products, after 24 h of *ergo* fermentation *E. coli* O157:H7 counts reached log 8.4 cfu/mL, but the mean counts decreased to log 6.47–6.58 cfu/mL when the milk was inoculated with LAB (Tsegaye & Ashenafi, 2005). The authors also reported that complete inactivation of *E. coli* O157:H7 was not achieved in the fermenting *ergo* and the count level was never below log 3 cfu/mL.

*Listeria monocytogenes*, a foodborne pathogen frequently detected in agricultural and food‐processing settings (Ulusoy & Chirkena, 2019), produces various virulence factors that can lead to both localized and systemic infections (Derra et al., 2013). This bacterium has become a significant concern in the dairy industry because of its capacity to endure a wide range of extreme conditions, including refrigeration temperatures and pH levels ranging from 4.3 to 10 (Yilma et al., 2007). *Listeria monocytogenes* is responsible for listeriosis in humans. Listeriosis outbreaks are frequently associated with dairy foods, including raw milk, ice cream, and soft cheese (CDC, 2004). Contamination, including post‐pasteurization contamination with *L. monocytogenes*, can be hazardous in food‐processing environments that do not implement good manufacturing practices, such as personal hygiene, equipment maintenance, training, and education. Foods refrigerated for longer than necessary can be a predisposing factor for the occurrence of *Listeria monocytogenes* (Ulusoy & Chirkena, 2019). Moreover, inadequate temperature and pasteurization are linked to *Listeria* occurrence. In Ethiopia, there are reports of the prevalence of *L. monocytogenes* in raw milk from different milk products raw milk (Derra et al., 2013), cottage cheese (Ashenafi, 1990), ice cream, yogurt, and pasteurized milk (Teshome et al., 2019). Derra et al. (2013) studied the occurrence of Listeria species in retail meat and milk products in Addis Ababa, Ethiopia, and revealed that 27.5% of the food samples tested were positive, and 4.1% of the isolates were identified as *L. monocytogenes*. In another study by Teshome et al. (2019), 30% of milk and milk products were positive for the presence of Listeria, 5% of which were *L. monocytogenes*.

Although *Salmonella* outbreaks are usually linked to poultry and beef products, dairy products such as raw milk and soft unpasteurized cheese, have also been implicated (Lobacz & Zulewska, 2021). *Salmonellae* have been reported in Ethiopia in various dairy products, including raw milk, butter, cheese, and yogurt (Ejo et al., 2016; Liyuwork et al., 2013; Mulaw and Tesfaye, 2017), *Ergo* and *Ayib* (Yilma et al., 2015). *Salmonella typhimurium* may survive for up to 72 h of fermentation and decline thereafter. Similarly, *Salmonella typhimurium* survived the cooking temperature of 50°C during *Ayib* making process (Yilma et al., 2015); complete destruction was achieved by cooking at 60 and 70°C. *Salmonella typhimurium* and *Salmonella enteritidis* have been observed in *Ergo* and butter because complete inhibition is not achieved within 24 h of fermentation (Yilma et al., 2007). *Ergo* is preferably consumed after 24 h of fermentation, owing to its desired flavor and taste. Moreover, reports have shown that the virulence of *Salmonella typhimurium* increases in acidic environments (pH 4.4–5.3) (Yilma et al., 2015). The most common source of *Salmonella* is the consumption of food that has been contaminated with animal feces or matter from the natural environment (Jajere, 2019). Liyuwork et al. (2013) studied the prevalence of and antimicrobial resistance profile of *Salmonella* isolates from milk products, including cheese, butter, and yogurt, and indicated that the overall prevalence was found to be 1.6%. In another study (Ejo et al., 2016), the overall prevalence of *Salmonella* was reported to be 5.5% for all food items tested including raw milk, raw meat, minced meat, burgers, and raw eggs.

*Staphylococcus aureus* is a common opportunistic pathogen in the dairy industry. It causes staphylococcal food poisoning owing to the production of enterotoxins (Deddefo et al., 2022). *Staphylococcus aureus* can survive in a wide range of environments, including dry, salty, and other stressful conditions (Tarekgne, 2016). *Staphylococcus aureus* has been isolated from different fermented milk products in Ethiopia, such as Ethiopian cottage cheese (Lemma et al., 2021), *ergo* (Zahara & Zinewi, 2015), *ayib* (Ashenafi, 2002), and *ititu* (Hussien et al., 2021). Deddefo et al. (2022) reported different pooled prevalence rates of *S. aureus* in various milk and milk products such as raw cow milk (30.7%), camel milk (19.3%), goat milk (13.6%), pasteurized milk (3.8%), Ethiopian cottage cheese (18.6%), and *ergo* (14.9%) in Ethiopia. Additionally, in traditionally prepared milk products, the overall prevalence rate of *S. aureus* was found to be 29.5% (Tarekgne, 2016). Similarly, Lemma et al. (2021) reported a prevalence rate of 24.6% for raw cow milk and 11.3% for traditionally prepared milk products including *ergo* and Ethiopian cottage cheese.

## 6 COMMERCIAL POTENTIAL OF TRADITIONAL FERMENTED MILK PRODUCTS OF ETHIOPIA

The dairy sector in Ethiopia contributes significantly (16.5%) to the national gross domestic product (GDP), employing 30% of the population (Duguma, 2022b). It is a source of income for smallholder farmers and is a readily available source of protein. However, dairy processing in the country remains underdeveloped owing to a number of challenges, including inadequate technical, institutional, policy, and socio‐economic constraints (Mihret et al., 2017).

Ethiopia's dairy sector is subsistence smallholder‐based and characterized by low production and productivity. It produces 4.69 billion liters of milk annually, of which 2.6% is processed in the milk processing industries (de Raad et al., 2021). Dairy products in Ethiopia are channeled to customers, through both formal and informal marketing systems. However, the informal marketing system is dominant, accounting 95% of the milk marketing in the country (Mebrate et al., 2020). In the formal market system, milk is collected at cooperative or private milk collection centers and delivered to processing plants, which distribute the products to consumers, caterers, supermarkets, and retailers (Abebe et al., 2014). This system encourages good hygienic practices for milk to avoid rejection upon delivery to collection centers. Therefore, producers supplying milk in this market system implement hygienic practices during production, storage, and transportation for acceptance. In an informal market system, producers directly sell milk products to consumers, unlicensed traders, or retailers by negotiating their prices (Yilma, 2012). The quality of milk products channeled through this system is poor, mainly because of limited knowledge of hygienic handling, inadequacy of dairy infrastructure, absence of milk quality tests, and other related facilities (de Raad et al., 2021).

Furthermore, fermented milk products such as *ergo* (naturally fermented Ethiopian milk), butter, ghee, and *ayib* (Ethiopian fresh cheese) are marketed through both formal and informal market channels. However, the majority of milk produced in rural areas is processed at the household level and marketed more through the informal market chain. Approximately, 44.6% of milk produced in Ethiopia is processed into butter and *ayib* (Ethiopian cottage cheese) (Bereda et al., 2020). The dairy market is mainly dominated by butter (36.6%) and *ayib* or Ethiopian cottage cheese (14.4%), particularly during the period of fasting where Ethiopian Orthodox Christians refrain from consuming milk; thus, the surplus amount is allowed to be processed into cheese and butter (Bereda et al., 2020). Smallholder‐marketed dairy products in Ethiopia do not have operational hygiene regulations. Some dairy products, such as *ayib*, are sold in the open market where they are exposed to further contamination and apparently affect the health of the consumer (Yilma, 2012). To develop a sustainable dairy production system, improving dairy supply through the scale‐up of traditional processing techniques is crucial, which in turn improves the marketing system of milk and milk products in Ethiopia. Large‐scale production could also help ensure its safety and nutrition while alleviating poverty and malnutrition.

## 7 CONCLUSION

Traditional fermented milk products in Ethiopia play a vital role in the daily diet of the population, and contribute to food, nutrition, and economic security. Their preparation is still at the household level and on a small scale, and the dairy industry is not well developed. Most Ethiopian fermented milk products are produced using traditional spontaneous and back‐slopping methods, relying on the fermentation of natural LAB and yeasts. Traditional fermented dairy foods lack consistent quality and safety because fermentation occurs under non‐controlled conditions. Therefore, to improve quality, safety, and production scale‐up, the use of a defined starter culture is necessary. Moreover, special attention on hygiene and handling of traditionally fermented products in Ethiopia is highly recommended if public health is to be guaranteed. It is essential to provide smallholder farmers with training and awareness of hygienic practices, such as washing and sanitizing hands before and after milking, cleaning and drying the udder with clean dry clothes, and proper washing of milk equipment. Additionally, efforts from both government and non‐governmental organizations are required to support good infrastructure with cooling facilities for milk collection and sale centers. Further studies involving meta‐analysis of fermented dairy products in Ethiopia and the region are recommended.

## AUTHOR CONTRIBUTIONS

**Karssa H. Tiruha:** Conceptualization (equal); data curation (equal); formal analysis (equal); investigation (equal); methodology (equal); resources (equal); software (lead); supervision (equal); validation (equal); visualization (equal); writing – original draft (lead); writing – review and editing (equal). **Jamal B. Kussaga:** Conceptualization (equal); data curation (equal); formal analysis (supporting); investigation (equal); methodology (equal); resources (equal); software (supporting); supervision (lead); validation (equal); visualization (equal); writing – original draft (supporting); writing – review and editing (equal). **Teresa Semedo‐Lemsaddek:** Conceptualization (equal); data curation (equal); formal analysis (supporting); investigation (equal); methodology (equal); resources (equal); software (supporting); supervision (lead); validation (equal); visualization (equal); writing – original draft (supporting); writing – review and editing (equal). **Jovin K. Mugula:** Conceptualization (equal); data curation (equal); formal analysis (supporting); investigation (equal); methodology (equal); resources (equal); software (supporting); supervision (lead); validation (equal); visualization (equal); writing – original draft (supporting); writing – review and editing (equal).

## FUNDING INFORMATION

This study was funded by the Partnership for Skills in Applied Sciences, Engineering, and Technology (PASET) through the Regional Scholarship and Innovation Fund (RSIF), FCT—Fundação para a Ciência e Tecnologia IP Portugal, through project PTDC/OCE‐ETA/1785/2020 [EMOTION], and Carnegie Corporation of New York (CCNY).

## CONFLICT OF INTEREST STATEMENT

The authors declare no conflict of interest.

## Data Availability

The datasets analyzed during the current study are available from the corresponding author upon reasonable request.
